# Epidemiologic transition and the double burden of disease in Ghana: What do we know at the neighborhood level?

**DOI:** 10.1371/journal.pone.0281639

**Published:** 2023-02-24

**Authors:** Irenius Konkor, Vincent Z. Kuuire

**Affiliations:** Department of Geography, Geomatics and Environment, University of Toronto Mississauga, Mississauga, Canada; Jazan University, SAUDI ARABIA

## Abstract

Many developing countries including Ghana are currently experiencing dual disease burdens emerging from an unprecedented risk overlap that drive their epidemiological transitions. Yet, siloed and disintegrated approaches continue to take precedence in health research and policy programs that drive competition for limited resources to address competing health problems. The objective of this study was to offer empirical evidence in support of a cogent argument for an integrated framework for the study and management of infectious and chronic health conditions in Ghana. We did so by examining the prevalence, determinants, and neighborhoods trajectories of the double burden of disease using data from a cross-sectional neighborhood-based study in Ghana. We fitted multinomial multilevel multivariate models to a sample of 1377 individual surveys and the results presented as odds ratios. Findings show that amidst a rising burden of NCDs, infectious diseases remain the most common health condition and participants in deprived neighborhoods were significantly more likely to report poor health outcomes. Risk factors such as tobacco and alcohol consumption were significantly associated with NCDs and infectious diseases and respondents who reported being diagnosed with NCDs and infectious diseases in the past year were likely to engage in leisure time physical activities and eat healthy. Based on our findings, we recommend health reforms in Ghana and argue for the design and implementation of an integrated framework for the study and management of the double burden of disease in Ghana and similar developing country settings.

## Introduction

Global historical health antecedents bordering on the dynamics of disease emergence particularly in industrialized countries led to the formulation of the epidemiologic transition model in the early 1970s [1–3]. Puchner and colleagues [4] argue that the epidemiologic transition model assumed noncommunicable and infectious diseases exact their heaviest burden territorially and that in settings where noncommunicable or infectious diseases thrive, the other was largely non-existent. Indeed, the epidemiologic transition model postulates that infectious diseases are gradually replaced by noncommunicable diseases (NCDs) together with shifts in mortality and morbidity toward older people. The generalizability of this assumption however later proved unsubstantiated in many geographic settings and especially so in low- and middle-income countries (LMICs). In 1990 for example, NCDs accounted for 27.79% of deaths in Ghana while infectious diseases (as well as maternal, neonatal, and nutritional diseases) were starkly responsible for 67.42% of all deaths [5]. By the end of 2019, however, deaths attributable to NCDs were over 46% while infectious diseases (and maternal, neonatal, and nutritional diseases] accounted for 47.17% [5]. Obviously, NCDs are not necessarily replacing infectious diseases in developing countries as is the case for Ghana but are instead coexisting. The dual burden of infectious diseases and NCDs within the same population has been described as the double burden of disease in the literature [6] and the objective of our study was to examine the prevalence, determinants, and neighborhoods trajectories of the double burden of disease in Ghana.

In describing the epidemiologic transition of LMICs using Mexico as the locus, Frenk et al. [7] made a passionate call for modifications of the epidemiologic transition model to accommodate health trends in developing countries. Of interest here was the call for recognition of a new epidemiological phase referred to as the *protracted epidemiologic transition* to describe a situation where shifts in ‘old’ health conditions do not entirely give way to ‘new’ diseases but instead coexist in the same population. After a comparative analysis of the causes of death at the population level in Mexico, Frenk et al. [7] described the situation as epidemiologic polarization because they observed a mixed pattern of deaths for the better-off in society and a pre-transitional pattern (infectious and nutrition-related causes) for the deprived in society. Thus, social class was observed to be a major determinant of the transition patterns. We believe that epidemiologic polarization best describes the current situation in Ghana caused by multiplicity of factors, and we argue researchers and health practitioners could adopt similar complex approaches to adequately respond to contemporary health needs in context.

### Epidemiologic transition in Ghana

A recent review of Ghana’s epidemiologic transition using archived data from the late 19^th^ to the 21^st^ century suggest Ghana has already undergone four main transitions and the latter of these transitions describes the situation as the double burden of disease [8]. Appiah-Agyekum [8] however described the transitions as a gradual reduction in tropical and infectious diseases in the face of a significant increase in NCDs. In an earlier study on the Epidemiologic transition of Accra (the most populous and urbanized city in Ghana) and motivated by the works of Frenk and colleagues, Agyei-Mensah and Aikins [9] described Ghana’s ongoing epidemiological phase as the *protracted polarized model*. They further noted a protracted double burden of infectious and chronic disease constitutes major cause of morbidity and mortality and that the wealthy group suffer higher risk of chronic diseases while residents in poor communities tend to suffer not only infectious disease burden but chronic health conditions as well. In both studies, the authors noted population growth and migration, colonization, urbanization, industrialization, commercialization, and globalization as key drivers of Ghana’s epidemiologic transitions. Unfortunately, however, the increase in NCDs was solely attributed to lifestyle changes linked to socio-economic development of the country despite the obvious contributions of environmental conditions to the growing burden of NCDs in Ghana [10].

Even though the double burden of disease is evident in Ghana, attempts at developing and implementing an integrated framework both in policy and in practice remain an unrealized endeavor. Among the numerous reasons, an integrated lens in the study and management of the double burden of disease is important because of the inherent clinical implications and related benefits. The so called big three infectious diseases (i.e., HIV, tuberculosis (TB), and malaria) can exacerbate the risk of developing some NCDs and can as well coexist with NCDs within the same individual. Puchner et al. [4] posit that 8 in ten people infected with mycobacterium TB would never have developed the disease but for NCDs (e.g., diabetes, obesity) induced compromised immune system and obstructive metabolic imbalance as well as increased stress that facilitate the manifestation of TB in newly infected people. Estimates from 2013 suggest 382 million people were diagnosed with diabetes mellitus in the world and was further projected to reach 642 million by 2040 with about 80% of these people living in LMICs [11, 12]. It is however suggested that diabetes mellitus increases the risk of being infected with diseases commonly caused by bacteria and fungi including TB, bloodstream, skin, feet and eye infections [12, 13]. In fact, some studies suggest people with diabetes are at 2–4 times increased risk of being infected with TB especially among those with poor glycemic control and the severity of such infection is higher than among non-diabetics [14]. Meanwhile, diabetes could impede antibiotic treatment, successful treatment of people with TB and facilitates relapse [15]. Others suggest preventive vaccinations might be less effective in diabetic people as proven with influenza vaccination outcome [16].

A study conducted in Tanzania has further revealed high rates of glucose metabolism disorders among HIV positive patients on antiretroviral medications in comparison to non-HIV positive people [17]. In South Africa and Ethiopia, empirical evidence has shown increased incidence of diabetes among people on long duration of HIV treatment [18, 19]. Other studies have also linked high rates of hepatitis C infection among diabetic patients living with HIV [20, 21]. In Ghana, Danquah and colleagues [22] conducted a case control study and observed asymptomatic malaria parasitaemia (plasmodium falciparum) was prevalent in people with type II diabetes than the control group and a unit increase in glucose concentration was found to increase the risk of malaria infection (plasmodium falciparum) by 5%. While NCDs have the potential to increase the risk of infectious disease and/or vice versa, there is the added potential complexity of complicating prognosis of either health conditions and especially so for infectious diseases. Chronic lung diseases can mimic TB imaging and symptoms which can obfuscate early diagnosis and thereby enhance further transmission within a population [4]. Sputum, and gastric fluid are important specimen for the diagnoses of infectious diseases such as TB. However, obtaining such specimen from NCD patients could be challenging especially when such patients have mental health conditions. As with TB and HIV, many NCDs require long-term medications. The interactive reaction between infectious disease and NCD medications may not only lead to poor treatment outcomes but can also create unique medical side effects.

While the double burden of disease and its related complications rage on in many LMICs, the traditional design of the healthcare system to respond to single episodes of care (e.g., TB, malaria, maternal health) still appear to be the dominant structure [23]. Similarly, researchers and health policy practitioners situated in LMICs and divided along lines of infectious and chronic diseases continue to fight for meagre resources to support their respective portfolios. Mohan and colleagues [24] argue that the siloed court for support is an artifact of self interest motivated by the individuals’ fields of specialization. Others however blame the epidemiologic transition model and how conservatives oversimplified it in order to artificially freeze social time to satisfy their own agendas [25]. The artificially freezing of social time, accordingly, enabled them to establish a before and after situations with the before representing the period of infectious disease burden and the after representing the “new” (i.e., NCDs) situation. Such a partition, they noted, made it easier for leadership to claim that one challenge (the infectious disease burden) had already been overcome while the NCDs burden is regarded as a pending issue–that might not require urgent attention.

Moreover, the traditionally disintegrated approaches within research and the healthcare systems often aim to achieve specific results within a given scale and time-frame at the expense of holistic health management strategies that are better placed at addressing health problems in a more comprehensive manner [26]. However, in resource constraint contexts where the double burden of disease phenomenon is pervasive, integrated approaches for the study and management of both conditions hold promise. As Temu et al. [26] cogently put it, at a time when the double burden of disease phenomenon is increasingly problematic, it is fundamental to use available limited resources in the most effective ways. Health planners, policy and decision makers must recognize that health systems can address issues of population health holistically and that the traditionally discrete approaches to the study and management of health conditions is long overdue [26]. In this study, we argue for a retooling of the healthcare system in Ghana and similar LMIC settings to effectively respond to the changing disease landscape and contemporary population health needs. To support the relevance of a new framework, we provide evidence that show the composite nature of Ghana’s disease profile.

### Methodological approaches

#### Study design and sampling

We designed a cross-sectional study to understand the connections between neighborhoods and health in Ghana. The study was implemented between the months of July and December 2021 in three cities i.e., Accra, Tamale and Wa which are divergent economically, development wise, and culturally and were selected for these reasons. Accra for example is the most developed, and administrative capital city of the republic. It is also the most populous city and believed to offer better economic opportunities which serve as a pull factor for migrants from different cultural backgrounds. Tamale on the other hand has been considered the fastest growing city in Ghana and has received major development facelift in recent years. The legacies of colonialism, subsequent and contemporary governmental neglects have left Wa as one of the most deprived administrative capital cities in the country and a reference point for poverty endemic indicators. The characteristics of these cities and their development dynamics are important for the emergence of certain health conditions.

For each city, neighborhoods were categorized into low-class, middle-class, and high-class residential areas and one was randomly selected from each category for the data collection (see upcoming work for details on how neighborhoods were defined). Households were subsequently systematically selected in each neighborhood and an adult member (18+ years) was randomly selected to respond to the survey. The survey asked questions on themes including history of chronic and infectious health conditions, access to and use of health services, health practices and risk behaviors, environmental characteristics and risk factors, and demographic and socio-economic characteristics of the respondents. No identifying information were collected. The sample size was computed using Cochrane’s sample size formula for cross-sectional study stated below:

$$n=\frac{Z^{2}pq}{e^{2}}$$

where n is the minimum required sample size, Z (1.96) is the alpha value corresponding to the 95% confidence interval, p (0.3) is the population prevalence of a variable of interest (NCDs), q is the difference of 1-p, and *e* (0.05) is the allowable error. Ethical clearance was obtained from the University of Toronto Social Sciences, Humanities and Education Research Ethics Board (*#00041065*), and the Navrongo Health Research Center, Ghana *(#App/NEHC/08/2021*). Written consent letter was read and interpreted to all participants and those who consented to participate went on to complete the survey.

#### Variable measures

The dependent variables were derived from two questions. In the survey, respondents were asked if a health professional had previously diagnosed them with any of the following infectious diseases; malaria, tuberculosis, cholera, respiratory infection, hepatitis, typhoid fever, food poisoning, HIV/AIDS, and/or COVID-19 in the 12 months prior the study. Responses were either yes or no for each health condition. Five infectious diseases (i.e., malaria, cholera, respiratory infection, hepatitis, and typhoid fever) had at least 1.5% prevalence in the study sample (see Table 2) and were combined to create the infectious disease variable coded as 0 = no or 1 = yes.

In a similar way, respondents were asked if a healthcare professional had previously diagnosed them with any of the following chronic health conditions: diabetes, hypertension, obesity, stroke, rheumatism, cancer, asthma, cardiovascular disease, chronic kidney disease, chronic liver disease, migraine, mental disorder, and/or other. We created the NCDs status variable by combing chronic health conditions (i.e., diabetes, hypertension, obesity, rheumatism, asthma, cardiovascular diseases, and/or migraine) with at least 1.5% (see Table 2) prevalence in the sample and coded it as 1 = yes for those who responded yes to any of these NCDs or 0 = no for those who responded no to being diagnosed with all the above chronic health conditions. We created the third and focal dependent variable (the double burden of disease) by combining the responses for both the NCDs and infectious diseases variables and we coded it as 0 = none (for participants who did not report any health condition), 1 = only NCDs (for participants who reported only NCDs), 2 = only infectious disease (for participants who reported only infectious diseases) and 3 = both (for participants who reported both infectious diseases and NCDs).

Because of our interest in further understanding the neighborhood dynamics of the double burden of disease in the study cities, we measured place-based variables including neighborhood aesthetic quality, neighborhood structural deprivation, neighborhood violence and neighborhood odor. Chum et al. [27] have cautioned against the use of respondents assessed neighborhood exposures in the study of health outcomes arguing that such approach contributes to the problem of same-source bias. To overcome this problem, they recommended averaging the responses of several residents in the same neighborhood so as to decouple the neighborhood exposure from the individual. Averaging over the measurement error in individual responses accordingly could produce a better measure of the neighborhood characteristics being studied. Hence, the individually assessed place-based variables in the survey were aggregated as collectively assessed neighborhood variables and standardized by transforming into z-scores.

Guided by the literature on known risk factors for both NCDs and infectious diseases, we further measured relevant variables including leisure time physical activity (LTPA), tobacco and alcohol consumption, weekly intake of fruits and vegetables, adding raw salt to meals at the dining table, how often respondent ate away from home in the previous week, and hygiene variables including access to toilet and tap water at home. The chronic health literature has largely focused on behavioral risk factors arguing that healthy diet, reduced alcohol, and tobacco consumption, and regular LTPA can reduce the risk of developing NCDs. Also, readily available infrastructure and utility services such as toilet and clean water at home can drastically reduce infectious disease transmissions and were measure for these purposes. We also measured variables on the socio-economic and demographic characteristics of the respondents such as age, gender, marital status, religion, educational level, employment status and wealth quintile.

#### Analytic strategies

To achieve the research objective, we conducted three separate but complementary analyses. The first was descriptive analysis to observe the distribution of the dependent and predictor variables. The second analysis was a multilevel binary complementary loglog analysis of the association between NCDs status, infectious disease status and the theoretically relevant predictor variables. For the third set of analysis, we built nested multinomial multilevel multivariate models of the relationship between the double burden of disease and the predictor variables. We considered the multinomial multilevel analysis appropriate because the dependent variable has four categories. Moreover, due to the stratified nature of the study design, the multilevel analytic technique was considered appropriate as it takes into account the different levels of stratifications.

Overall, three nested models were built. We accounted for place-based variables including neighborhood aesthetic quality, neighborhood structural deprivation, neighborhood violence, neighborhood odor, time spent in neighborhood in a typical week, and length of stay in current residential neighborhood in model 1. Accounting for time spent in neighborhood on weekly basis and length of stay in current residential neighborhood were considered particularly important because of the possibility of neighborhood mobility and the un-contemporaneous nature of chronic health conditions. Thus, exposure to health risk factors might have happened at a previous location and time and the manifestation of the resultant health conditions at another time and location. Ignoring such crucial potential confounders in place-based analysis could therefore produce misleading results and contribute to the uncertain geographic context problem. In the second model, we accounted for risk factors for both infectious diseases and NCDs such as access to clean water and toilet facilities at home, fruits, and vegetable consumption, eating away from home, salt consumption, LTPA, tobacco use, and alcohol consumption. We further accounted for socio-economic and demographic characteristics of the respondents in the third model. We used Stata software version 14.2 for all statistical analyses and the results were exponentiated and presented as odds ratios (ORs).

## Results

### Descriptive results

As shown in column 2 Table 1. the average age of respondents was 41.7 years and about half (51.2%) identified as female. A higher proportion of the sample reported being married (62%) and 62.6% identified with the Christian faith. A little more than a third (36.6%) attained tertiary education and about half (51.5%) were privately employed and wealth quintile was relatively evenly distributed. More than a fifth (21.7%) of respondents had lived in their current residential neighborhoods for over 20 years and about 43.8% would often spend the entire week in their residential neighborhoods. Respondents overall rated the aesthetic quality of their neighborhoods above average (24.9/40), and so was neighborhood structural deprivation (26.4/40) and neighborhood odor (12.0/20). Neighborhood level violence however was rated below average (10.5/24). About a third (33%) of respondents reported consuming four or more servings of fruits and nearly half (49%) consumed four or more servings of vegetables in the week prior the interview. More than a fifth (21.4%) would often add raw salt to their meals at the dining table when they felt the salt was not enough and 13.5% ate away from home everyday in the week prior the interview. Nearly 1 in 10 participants (9.2%) reported using tobacco products in the past and about a quarter (23.1%) reported consuming alcohol and leisure time physical inactivity was relatively high (30.2%). Interestingly, nearly half (47.4%) did not have toilet (water closet) at home and about a third (31.8%) did not have access to tap water at home.

**Table 1:** Descriptive statistics and bivariate results of the relationship between the dependent and independent variables.

Variable	Descriptive results	Bivariate results	
		NCDs models	Infectious disease models
	Percent/mean(Freq.)	OR[95%CI]	OR[95%CI]
**Neighborhood aesthetic quality** †	24.9	0.98[0.84,1.15]	0.85[0.76,0.94]**
**Neighborhood structural deprivation** †	12.0	0.96[0.83,1.11]	0.95[0.85,1.07]
**Neighborhood violence** †	26.4	0.85[0.74,0.97]*	---------------
**Neighborhood odor** †	10.5	1.05[0.93,1.18]	1.02[0.94,1.12]
**Time in neighborhood**			
Mostly away	11.2(155)	1.00[1.00,1.00]	1.00[1.00,1.00]
A few days	16.6(229)	0.82[0.52,1.30]	0.77[0.57,1.03]
Most days	28.4(392)	1.17[0.79,1.74]	0.70[0.53,0.92]*
Entire week	43.8(606)	1.70[1.16,2.48]**	0.82[0.63,1.06]
**Length in neighborhood**			
1–5	22.4(310)	1.00[1.00,1.00]	1.00[1.00,1.00]
6–10	19.0(263)	1.18[0.74,1.86]	0.89[0.71,1.12]
11–15	11.0(152)	2.64[1.70,4.10]***	0.92[0.71,1.20]
16–20	8.5(118)	2.15[1.32,3.51]**	1.03[0.78,1.37]
20+	21.7(300)	3.49[2.38,5.13]***	0.70[0.56,0.88]**
Entire life	17.4(240)	3.44[2.23,5.32]***	1.35[1.04,1.74]*
**Fruits consumption**			
1 serving	16.2(223)	1.00[1.00,1.00]	1.00[1.00,1.00]
2 servings	20.0(276)	0.74[0.52,1.05]	0.97[0.76,1.24]
3 servings	18.6(257)	0.67[0.47,0.97]*	1.20[0.94,1.55]
4+ servings	33.0(456)	0.87[0.63,1.21]	1.18[0.93,1.49]
Other	12.2(169)	0.91[0.61,1.37]	0.72[0.54,0.96]*
**Vegetable consumption**			
1 serving	9.8(135)	1.00[1.00,1.00]	1.00[1.00,1.00]
2 servings	13.7(189)	0.97[0.63,1.50]	0.79[0.59,1.07]
3 servings	17.9(248)	0.67[0.43,1.04]	0.86[0.65,1.15]
4+ servings	49.0(677)	0.71[0.48,1.05]	0.88[0.68,1.14]
Other	9.6(133)	1.04[0.65,1.68]	0.63[0.45,0.88]**
**Salt at dining table**			
No	78.6(1087)	1.00[1.00,1.00]	---------------
Yes	21.4(296)	0.85[0.65,1.13]	---------------
**Eat away from home**			
Not at all	13.5(186)	1.00[1.00,1.00]***	1.00[1.00,1.00]
Two and below	20.8(287)	0.47[0.36,0.61]***	1.01[0.85,1.21]
Three or more	36.5(506)	0.56[0.42,0.77]***	1.17[0.95,1.44]
Everyday	29.2(404)	0.42[0.29,0.62]***	0.81[0.61,1.07]
**Tobacco use**			
No	90.8(1256)	1.00[1.00,1.00]	---------------
Yes	9.2(127)	3.04[2.28,4.05]***	---------------
**Alcohol consumption**			
No	76.9(1064)	1.00[1.00,1.00]	---------------
Yes	23.1(319)	0.88[0.67,1.15]	---------------
**LTPA**			
Never	30.2(417)	1.00[1.00,1.00]	---------------
Rarely	31.5(437)	0.81[0.61,1.07]	---------------
1–2 times	21.4(297)	0.75[0.55,1.04]	---------------
3+ times	16.9(233)	0.99[0.72,1.36]	---------------
**Tap water at home**			
Yes	68.2(943)	---------------	1.00[1.00,1.00]
No	31.8(440)	---------------	1.06[0.89,1.25]
**Toilet at home**			
Yes	52.6(728)	---------------	1.00[1.00,1.00]
No	47.4(655)	---------------	0.86[0.73,1.02]
**Educational level**			
Tertiary education	36.7(507)	1.00[1.00,1.00]	1.00[1.00,1.00]
Secondary education	29.9(414)	0.80[0.59,1.09]	0.95[0.78,1.16]
Primary education	19.2(266)	1.11[0.78,1.57]	1.12[0.89,1.42]
No formal education	14.2(196)	2.70[1.94,3.74]***	1.12[0.87,1.44]
**Employment status**			
Privately employed	51.5(712)	1.00[1.00,1.00]	1.00[1.00,1.00]
Government employed	14.6(202)	0.71[0.50,1.02]	1.33[1.06,1.66]*
Unemployed	12.1(167)	1.60[1.14,2.24]**	1.15[0.90,1.47]
Other	21.8(302)	0.90[0.68,1.19]	1.16[0.96,1.40]
**Wealth quintile**			
Poorest	20.1(279)	1.00[1.00,1.00]	1.00[1.00,1.00]
Poorer	20.1(278)	0.76[0.53,1.09]	1.01[0.80,1.27]
Middle	19.7(272)	0.78[0.55,1.12]	0.84[0.66,1.07]
Richer	20.4(282)	0.85[0.59,1.23]	1.15[0.90,1.47]
Richest	19.7(272)	1.15[0.77,1.73]	1.01[0.76,1.36]
**Age** †	41.7	1.05[1.05,1.06]***	1.00[1.00,1.01]
**Gender**			
Male	48.8(675)	1.00[1.00,1.00]	1.00[1.00,1.00]
Female	51.2(708)	1.13[0.91,1.40]	1.26[1.09,1.47]**
**Marital status**			
Single	19.1(264)	1.00[1.00,1.00]	1.00[1.00,1.00]
In relationship	7.5(104)	0.68[0.32,1.42]	0.87[0.63,1.19]
Married	62.0(857)	2.26[1.57,3.27]***	1.02[0.84,1.23]
Divorced/Widowed	11.4(157)	4.99[3.31,7.54]***	1.02[0.77,1.34]
**Religion**			
Christian	62.6(866)	1.00[1.00,1.00]	1.00[1.00,1.00]
Muslim	35.9(497)	1.33[1.02,1.72]*	1.03[0.85,1.25]
Other	1.5(20)	1.25[0.55,2.83]	1.31[0.73,2.34]

*P<0.05,

**P<0.01,

***P<0.001,

^**†**^ **=** mean reported,

CI = confidence interval. Note: For ease of interpretation, descriptive neighborhood variables were presented as summative averages.

As shown in Table 2 below the commonly reported infectious disease was malaria (56.8%) while the most reported NCD was hypertension (13.1%). About 18% of the sample were diagnosed with both chronic and infectious diseases and 7.6% reported being diagnosed with only NCDs. A higher proportion (44.6%) indicated being diagnosed with only infectious diseases in the 12 months prior the study and a little less than a third (30.1%) did not report being diagnosed with any health condition.

**Table 2:** Distribution of infectious diseases and chronic health conditions.

Health condition	Distribution
Infectious diseases	Prevalence rate in percent (Freq.)
Malaria	Yes = 56.8
	No = 43.2
Cholera	Yes = 1.7
	No = 98.3
Respiratory infection	Yes = 11.1
	No = 88.9
Hepatitis	Yes = 1.7
	No = 98.3
Typhoid fever	Yes = 10.8
	No = 89.2
**NCDs**	
Diabetes	Yes = 5.7
	No = 94.3
Hypertension	Yes = 13.1
	No = 86.9
Obesity	Yes = 1.5
	No = 98.5
Rheumatism	Yes = 2.0
	No = 98.0
Asthma	Yes = 2.2
	No = 97.8
Cardiovascular diseases,	Yes = 1.5
	No = 98.5
Migraine	Yes = 4.9
	No = 95.1
**Double burden of disease**	
None	30.1(414)
Only NCDs	7.6(105)
Only infectious disease	44.6(614)
Both	17.7(244)

### Bivariate results

First, we conducted multilevel binary complementary loglog analysis of the relationship between NCDs status and the predictor variables and the results are presented in Table 1 column 3. We observed that respondents who typically spent the entire week in their current residential neighborhood were 1.7 times more likely to be diagnosed with NCDs compared to those who were mostly away. Compared to respondents who have been living in their current neighborhood for less than 6 years, those who have been living there for 11–15 years [OR = 2.64, CI = 1.70,4.10], 16–20 years [OR = 2.15, CI = 1.32,3.51], 20+ years [OR = 3.49, CI = 2.38,5.13] and entire life [OR = 3.44, CI = 2.23,5.32] were more likely to be diagnosed with NCDs. A standard deviation increase in neighborhood violence was observed to be associated with lower likelihood of being diagnosed with NCDs. Consuming 3 servings of fruits in the previous week was observed to be significantly associated with lower likelihood of being diagnosed with NCDs but tobacco users were more likely [OR = 3.04, CI = 2.28,4.05] to report being diagnosed with NCDs. Respondents who did not receive formal education [OR = 2.70, CI = 1.94,3.74] and those who were unemployed [OR = 1.60, CI = 1.14,2.24] were more likely to report being diagnosed with NCDs. A year increase in age was observed to be significantly associated with 5% higher likelihood [OR = 1.05, CI = 1.05,1.06] of being diagnosed with NCDs. Compared with the never married, married respondents [OR = 2.26, CI = 1.57,3.27], and divorced/widowed [OR = 4.99, CI = 3.31,7.54] respondents were more likely to be diagnosed with NCDs. Muslims were also more likely [OR = 1.33, CI = 1.02,1.72] to report being diagnosed with NCDs compared with Christians.

We also conducted multilevel binary complementary loglog analysis of the relationship between infectious disease status and the predictor variables and the results are presented in column 4 Table 1. We noticed remarkable differences from the NCDs results. For example, unlike the NCDs results, we found that a standard deviation increase in neighborhood aesthetic quality was significantly associated with lower [OR = 0.85, CI = 0.76,0.94] likelihood of reporting being diagnosed with infectious disease in the twelve months prior the study. Compared with respondents who were mostly away from their residential neighborhoods, those who were there most days of the week were less likely [OR = 0.70, CI = 0.53,0.92] to report being diagnosed with infectious disease. Participants who spent 20+ years in their current residential neighborhood were less likely [OR = 0.70, CI = 0.56,0.88] to report being diagnosed with infectious disease in the previous year but those who had lived in their current residential neighborhoods all their lives were more likely [OR = 1.35, CI = 1.04,1.74] to be diagnosed with infectious disease. Respondents who ate 3 or more times away from home in the previous week prior the study were 1.4 times likely to be diagnosed with infectious disease. Women were also more likely [OR = 1.26, CI = 1.09,1.47] to report being diagnosed with infectious disease compared with men. Compared with the privately employed respondents, government employees were 1.3 times more likely [OR = 1.33, CI = 1.06,1.66] to report being diagnosed with infectious disease.

### Multivariate results

To further examine the factors associated with the double burden of disease, we conducted multinomial multilevel multivariate analysis and the results of the final model (i.e., model 3) are presented in Table 3 (see S1 Appendix for full nested models). It is interesting to note the results did not show uniform pattern on the variables that are significant determinants of the three health outcomes (i.e., only NCDs, only infectious disease or both) after accounting for all theoretically relevant variables. For instance, relative to none of the health conditions, a standard deviation increase in neighborhood structural deprivation was observed to be significantly associated with higher likelihood [AOR = 1.61, CI = 1.08,2.41] of reporting being diagnosed with NCDs but not with infectious disease. On the other hand, a standard deviation increase in neighborhood aesthetic quality predicted lower likelihood [AOR = 0.73, CI = 0.58,0.92] of reporting being diagnosed with only infectious disease in the past year. However, the likelihood of reporting being diagnosed with both NCDs, and infectious disease was higher [AOR = 1.27, CI = 1.03,1.57] for participants residing in neighborhoods characterized with elevated levels of odor. Compared with respondents who were mostly away from their residential neighborhood, those who were there a few days a week [AOR = 0.52, CI = 0.28,0.98], or most days of the week [AOR = 0.45, CI = 0.24,0.82] were less likely to report being diagnosed with infectious disease. Participants who spent their entire life in one neighborhood were significantly more likely to be diagnosed with only NCDs [AOR = 4.72, CI = 1.57,14.17], or both [AOR = 4.51, CI = 2.17,9.33]. For infectious disease, however, staying in current residential neighborhood over 20 years was significantly associated with lower likelihood [AOR = 0.53, CI = 0.34,0.83] of reporting being diagnosed positive. Consuming 3 servings of fruits [AOR = 1.73, CI = 1.03,2.91], and 4 servings of vegetables [AOR = 3.14, CI = 1.01,9.75] in the previous week were observed to be significantly associated with increased likelihood of reporting being diagnosed with infectious disease and NCDs respectively. Tobacco users however were more likely to be diagnosed with both NCDs and infectious disease [AOR = 2.26, CI = 1.20,4.26], but alcohol consumption [AOR = 1.55, CI = 1.06,2.26] was significantly associated with only infectious disease.

**Table 3:** Multinomial multilevel multivariate analysis of the relationship between chronic and/or infectious disease status and theoretically relevant variables.

Variable	NCDs only	Infectious disease only	Both
	AOR [95% CI]	AOR [95% CI]	AOR [95% CI]
**Neighborhood aesthetic quality**	0.91[0.62,1.34]	0.73[0.58,0.92]**	0.79[0.59,1.06]
**Neighborhood structural deprivation**	1.61[1.08,2.41]*	1.11[0.88,1.41]	1.11[0.82,1.48]
**Neighborhood violence**	1.21[0.92,1.59]	0.97[0.82,1.14]	0.83[0.66,1.04]
**Neighborhood odor**	1.15[0.87,1.51]	1.07[0.91,1.26]	1.27[1.03,1.57]*
**Time in neighborhood**			
Mostly away	1.00[1.00,1.00]	1.00[1.00,1.00]	1.00[1.00,1.00]
A few days	0.78[0.25,2.45]	0.52[0.28,0.98]*	0.51[0.23,1.15]
Most days	0.76[0.26,2.22]	0.45[0.24,0.82]**	0.51[0.24,1.09]
Entire week	0.76[0.26,2.21]	0.55[0.30,1.01]	0.48[0.23,1.01]
**Length in neighborhood**			
1–5	1.00[1.00,1.00]	1.00[1.00,1.00]	1.00[1.00,1.00]
6–10	1.01[0.38,2.71]	0.87[0.58,1.30]	0.64[0.32,1.26]
11–15	1.92[0.69,5.34]	0.85[0.50,1.45]	1.17[0.56,2.44]
16–20	0.99[0.30,3.25]	1.08[0.60,1.93]	1.04[0.47,2.32]
20+	1.59[0.62,4.03]	0.53[0.34,0.83]**	1.02[0.54,1.92]
Entire life	4.72[1.57,14.17]**	1.69[0.95,3.02]	4.51[2.17,9.33]***
**Fruits consumption**			
1 serving	1.00[1.00,1.00]	1.00[1.00,1.00]	1.00[1.00,1.00]
2 servings	0.51[0.20,1.28]	1.07[0.66,1.75]	0.73[0.39,1.37]
3 servings	0.97[0.37,2.50]	1.73[1.03,2.91]*	1.27[0.64,2.50]
4+ servings	0.88[0.37,2.10]	1.62[0.99,2.66]	1.27[0.68,2.39]
Other	1.23[0.43,3.52]	0.96[0.54,1.69]	0.61[0.28,1.33]
**Vegetable consumption**			
1 serving	1.00[1.00,1.00]	1.00[1.00,1.00]	1.00[1.00,1.00]
2 servings	2.59[0.77,8.68]	0.70[0.39,1.25]	0.72[0.34,1.51]
3 servings	2.38[0.69,8.20]	0.91[0.52,1.61]	0.83[0.40,1.76]
4+ servings	3.14[1.01,9.75]*	0.91[0.53,1.55]	0.69[0.35,1.37]
Other	1.86[0.45,7.66]	0.55[0.28,1.09]	1.20[0.51,2.86]
**Salt at dining table**			
No	1.00[1.00,1.00]	1.00[1.00,1.00]	1.00[1.00,1.00]
Yes	1.84[0.96,3.55]	1.55[1.07,2.25]*	1.20[0.73,1.99]
**Eat away from home**			
Not at all	1.00[1.00,1.00]	1.00[1.00,1.00]	1.00[1.00,1.00]
Two and below	0.57[0.31,1.08]	0.98[0.68,1.43]	0.65[0.40,1.07]
Three or more	1.02[0.48,2.19]	1.50[0.97,2.33]	1.50[0.85,2.67]
Everyday	0.25[0.07,0.88]*	0.61[0.35,1.07]	0.82[0.40,1.66]
**Tobacco consumption**			
No	1.00[1.00,1.00]	1.00[1.00,1.00]	1.00[1.00,1.00]
Yes	1.86[0.87,3.99]	0.77[0.42,1.39]	2.26[1.20,4.26]*
**Alcohol consumption**			
No	1.00[1.00,1.00]	1.00[1.00,1.00]	1.00[1.00,1.00]
Yes	0.95[0.50,1.82]	1.55[1.06,2.26]*	1.41[0.84,2.35]
**LTPA**			
Never	1.00[1.00,1.00]	1.00[1.00,1.00]	1.00[1.00,1.00]
Rarely	2.20[1.11,4.38]*	2.24[1.54,3.26]***	0.97[0.59,1.61]
1–2 times	1.32[0.59,2.96]	2.17[1.40,3.35]***	1.26[0.71,2.22]
3+ times	2.56[1.08,6.05]*	3.02[1.83,4.99]***	2.51[1.35,4.65]**
**Tap water at home**			
Yes	1.00[1.00,1.00]	1.00[1.00,1.00]	1.00[1.00,1.00]
No	3.00[1.19,7.56]*	1.65[1.03,2.64]*	1.99[1.02,3.88]*
**Toilet at home**			
Yes	1.00[1.00,1.00]	1.00[1.00,1.00]	1.00[1.00,1.00]
No	0.28[0.11,0.71]**	0.64[0.39,1.05]	0.73[0.37,1.44]
**Educational level**			
Tertiary education	1.00[1.00,1.00]	1.00[1.00,1.00]	1.00[1.00,1.00]
Secondary education	0.41[0.19,0.90]*	1.16[0.76,1.76]	0.93[0.51,1.68]
Primary education	0.43[0.16,1.13]	1.47[0.87,2.47]	1.38[0.68,2.79]
No formal education	0.67[0.24,1.92]	1.51[0.79,2.91]	1.32[0.59,2.92]
**Employment status**			
Privately employed	1.00[1.00,1.00]	1.00[1.00,1.00]	1.00[1.00,1.00]
Government employed	0.33[0.13,0.82]*	1.27[0.77,2.09]	1.01[0.51,1.99]
Unemployed	1.22[0.51,2.93]	0.96[0.58,1.58]	1.07[0.56,2.05]
Other	0.41[0.20,0.84]*	1.28[0.88,1.88]	0.74[0.44,1.25]
**Wealth quintile**			
Poorest	1.00[1.00,1.00]	1.00[1.00,1.00]	1.00[1.00,1.00]
Poorer	1.96[0.70,5.48]	1.23[0.75,2.02]	1.14[0.57,2.28]
Middle	1.68[0.45,6.29]	0.85[0.45,1.62]	1.07[0.42,2.70]
Richer	0.69[0.14,3.45]	1.18[0.54,2.60]	1.99[0.65,6.09]
Richest	0.90[0.16,5.00]	0.69[0.28,1.70]	1.53[0.44,5.34]
**Age**	1.07[1.05,1.10]***	1.01[0.99,1.02]	1.07[1.05,1.09]***
**Gender**			
Male	1.00[1.00,1.00]	1.00[1.00,1.00]	1.00[1.00,1.00]
Female	1.89[1.04,3.44]*	1.84[1.32,2.57]***	2.90[1.85,4.53]***
**Marital status**			
Single	1.00[1.00,1.00]	1.00[1.00,1.00]	1.00[1.00,1.00]
In relationship	1.78[0.48,6.60]	0.92[0.52,1.63]	0.41[0.12,1.35]
Married	1.11[0.41,2.99]	1.03[0.66,1.61]	0.80[0.42,1.52]
Divorced/Widowed	1.14[0.33,3.97]	0.80[0.39,1.64]	0.86[0.35,2.09]
**Religion**			
Christian	1.00[1.00,1.00]	1.00[1.00,1.00]	1.00[1.00,1.00]
Muslim	0.97[0.50,1.90]	1.15[0.77,1.72]	1.39[0.84,2.31]
Other	0.81[0.11,6.03]	2.34[0.60,9.13]	0.98[0.21,4.64]

*P<0.05,

**P<0.01,

***P<0.001,

^**†**^ **=** mean reported,

CI = confidence interval

Consuming meals prepared outside the home every day in the previous week was observed to be significantly associated with lower likelihood of reporting NCDs positive status. Engaging in leisure time physical activity 3 or more times per week was observed to be associated with increased likelihood of reporting only NCDs [AOR = 2.56, CI = 1.08,6.05], only infectious disease [AOR = 3.02[1.83,4.99], and both [AOR = 2.51, CI = 1.35,4.65]. Participants who did not have access to tap water at home were similarly more likely to report only infectious disease [AOR = 1.65, CI = 1.03,2.64], only NCDs [AOR = 3.00, CI = 1.19,7.56], or both [AOR = 1.99, CI = 1.02,3.88]. The socio-demographic and economic characteristics show respondents who attained up to secondary education [AOR = 0.41, CI = 0.19,0.90] and those who were government employees [AOR = 0.33, CI = 0.13,0.82] were less likely to report being diagnosed with NCDs compared to those who attained up to tertiary education or were privately employed respectively. The likelihood of reporting being diagnosed with infectious disease did not significantly differ by age. However, a year increase in age was found to be associated with a 7% higher risk of only NCDs [AOR = 1.07, CI = 1.05,1.10], or both [AOR = 1.07, CI = 1.05,1.09]. Identifying as female was unfortunately observed to be significantly associated with increased likelihood of reporting being diagnosed with only NCDs [AOR = 1.89, CI = 1.04,3.44], only infectious disease [AOR = 1.84, CI = 1.32,2.57], or both [AOR = 2.90, CI = 1.85,4.53].

## Discussion and conclusions

The findings show that amidst a rising burden of NCDs, infectious diseases (i.e., malaria) remain the most common health condition in the study setting. About a quarter of the sample reported NCDs positive status and nearly a fifth reported suffering a brunt of the double burden of disease. Although the place-based factors relevant for health outcomes were not uniformly significant for all three health outcomes measured, the observed pattern was that residents in deprived neighborhoods were significantly more likely to report poor health outcomes.

The negative relationship between neighborhood deprivation and poor health outcomes demonstrates the important contributions of place on the double burden of disease in Ghana where differential neighborhood environments is a manifestation of widening wealth disparities. Urbanization of African cities has been noted to be largely demographic in outlook and therefore does not commensurate with the economic and infrastructural development of the continent [28]. The slow pace of economic and infrastructural developments in urban settings consequently contribute to high rates of unemployment, low wages, poverty, and inequalities that have implications for poor health outcomes [29]. Indeed, plethora of research evidence has drawn the connections between high morbidity and mortality rates among residents in deprived neighborhoods compared to residents of the more affluent neighborhoods even after accounting for the individual level characteristics [30–32]. Poor city planning and/or the lack of enforcement of planned building codes commonly reflected in poor drainage systems across cities in Ghana is a source of noxious stench/odor and breeding grounds for disease causing pathogens including mosquitoes and vibrio cholerae bacteria that cause malaria and cholera respectively. Moreover, poor urban built forms contribute to lack of activity spaces and poor ventilation that exacerbate NCD risk factors such as air pollution and second-hand smoking in deprived neighborhoods. In fact, our findings support the understanding that tobacco consumption increases the risk of being diagnosed with NCDs. Algren and colleagues [31] in a similar study found elevated levels of smoking and physical inactivity among people living in deprived neighborhoods compared to those in affluent neighborhoods. However, it is important to point out the effects of neighborhood environments on health outcomes is not contemporaneous and especially so for chronic health conditions due to the latency period of many chronic illnesses. That notwithstanding, we still found significant associations between neighborhood deprivation and poor health outcomes even after accounting for the effect of time spent in neighborhood on weekly basis and length of stay in current residential neighborhood.

Preventative science literature unequivocally argues for healthy eating including the consumption of fruits and vegetables as a healthy practice for minimizing infectious and chronic health risks [33, 34]. It is however interesting to note that increased consumption of fruits and vegetables were observed to be associated with increased likelihood of infectious disease and NCDs respectively. Another apparently counter-intuitive observation was that engaging in regular weekly leisure time physical activity was associated with higher odds of all 3 health outcomes. A cluster-randomized trial study on culturally adapted intervention education elsewhere for example found adherence to lifestyle recommendations about physical activity, nutrition, smoking, and/or alcohol consumption were higher among patients who received culturally sensitive health education compared with those who did not receive it [35]. Moreover, unlike healthy people, unhealthy peoples’ dietary choices and physical activities might be conditioned by their health conditions and informed by the recommendations of their physicians. In the Ghanaian context, earlier studies found illness perceptions and diabetes knowledge among type II diabetes patients influenced their healthy dietary choices and physical activities [36]. It is therefore not surprising that respondents who reported being diagnosed with NCDs, infectious disease or both would be inclined to healthy practices including eating well and routinely exercising.

One other interesting finding from our analysis is that, unlike infectious diseases, age was found to be significantly associated with NCDs status. This is consistent with previous studies that observed higher burden of NCDs among older Ghanaian [37, 38]. While older age might be an important determinant of the growing burden of NCDs, social inequities could be the invisible driving force [39]. As demonstrated in our results, elements of societal inequities contributed significantly to the patterns of NCDs and the double burden of disease more broadly. For instance, access to clean water is vital for minimizing infectious diseases through improved hygiene and regular handwashing yet, tap water was not readily available to every household. In fact, having access to tap water at home in the Ghanaian context is an indicator of higher social status and only households that have attained such status can afford it. What is even worrying and consistent with previous studies [40], we found gender variations in the double burden of disease as women were observed to be at increased risk of being diagnosed with NCDs, infectious disease or both. This observation further highlights underlying structural factors that perpetuate gender and societal inequities.

Even though the findings from our analysis are insightful there may be some limitations associated with our study worth highlighting. The first and most important limitation of these is the self-reported nature of the variables measured. Unlike acute and episodic health conditions, NCDs develop very slowly and are very difficult to detect especially in situations where people do not screen periodically. In fact, previous studies in Ghana have found higher actual measure prevalence of chronic diseases (i.e., hypertension) than self-reported chronic disease prevalence [37, 41]. It is therefore probable the NCDs prevalence rate in our sample could be different than the actual prevalence. Same-source bias has also arisen as a major concern in place-based studies with proponents arguing that people with poor health outcomes are often likely to report poor neighborhood indicators. To overcome this challenge in the absence of objectively measured neighborhood variables, Chum and colleagues [27] recommended averaging over the individual responses so as to decouple reports of the reported neighborhood exposures from the individual. Consequently, the place-based measures were aggregated and transformed into z-scores to produce standardized and comparative scales. Finally, our analysis was based on cross-sectional data and the results do not imply causation.

Notwithstanding the above limitations, the siloed approach to the study and practices within the healthcare system is overdue and there is the urgent need to integrate the two health conditions in the healthcare system and in research rather than continue to compete for limited resource to respond to one health condition or the other. There must be adequate understanding of the extend of the problem among health stakeholders and relevant partners to be able to design any effective integrated framework. Even though further research may be needed in this regard, an integrated framework must be community oriented with well defined population prevention strategies, surveillance and reporting mechanisms, early diagnostics, and treatment protocols [42]. Moreover, since health transition could manifest incongruently within the same nation, the integrated framework could incorporate scale lens–micro, meso, and macro levels. Irrespective of the level, the framework should coordinate with other sectors including environmental and urban planners, housing and recreation and other sectors relevant for improving the overall health and wellbeing of the population.

Finally, policy discussions on health systems design often need to recognize two key elements– 1) understand the prevailing health conditions/needs of the population in question, and 2) identify the appropriate responses that must be implemented in response to these needs [7] using the available limited resources. The evidence from this study shows that understanding the former requires the double burden of disease lens as it produces a more holistic population health profile of Ghana. Based on our understanding of the lag period between exposure to health risk factors and the subsequent manifestation of the resultant diseases particularly NCDs, we argue that many of the chronic health patients that will require the services of the healthcare system within a decade or two from now have already been exposed. While tremendous gains have been made regarding curtailing infectious diseases, the expectation is that they will continue to linger for awhile conditioning the persistence of a protracted burden of chronic and infectious disease. This emerging concern will affect many sectors including the health system and even families. Within the healthcare system for instance, the *no bed syndrome* in hospitals in Ghana could get worse as a manifestation of this emerging phenomenon since many NCDs, unlike episodic and acute diseases, require long-term care. There could also be dire consequences for families both financially and socially as patients will require the services and support of their relatives. Any effective policy intervention must as a matter of urgency factor in these pressing concerns.

## Supporting information

S1 Appendix (DOCX)
