# Supplementary material for: Epidemiologic transition and the double burden of disease in Ghana: What do we know at the neighborhood level?
Source: PLoS One. 2023 Feb 24;18(2):e0281639. doi: 10.1371/journal.pone.0281639 (PMC9956066; doi:10.1371/journal.pone.0281639)
Supplement: S1 Appendix — (DOCX) [file pone.0281639.s001.docx]

**S1 Appendix**

Table 4. Multinomial multilevel multivariate analysis of the relationship between chronic and/or infectious disease status and theoretically relevant variables

|  | NCDs only | Infectious disease only | Both |
| --- | --- | --- | --- |
|  | RRR [95% CI] | RRR [95% CI] | RRR [95% CI] |
| **Model 1** | | | |
| **Neighborhood aesthetic quality** | 0.90[0.66,1.23] | 0.61[0.50,0.76]^***^ | 0.71[0.55,0.91]^**^ |
| **Neighborhood structural deprivation** | 1.27[0.92,1.75] | 1.09[0.89,1.35] | 1.06[0.83,1.36] |
| **Neighborhood violence** | 1.02[0.80,1.29] | 1.00[0.86,1.16] | 0.72[0.59,0.88]^**^ |
| **Neighborhood odor** | 0.99[0.79,1.25] | 1.10[0.94,1.27] | 1.21[1.00,1.45] |
| **Time in neighborhood** |  |  |  |
| Mostly away | 1.00[1.00,1.00] | 1.00[1.00,1.00] | 1.00[1.00,1.00] |
| A few days | 0.74[0.26,2.06] | 0.56[0.31,1.01] | 0.48[0.23,1.01] |
| Most days | 0.81[0.32,2.07] | 0.44[0.25,0.76]^**^ | 0.49[0.25,0.96]^*^ |
| Entire week | 1.24[0.50,3.07] | 0.61[0.35,1.04] | 0.83[0.44,1.59] |
| **Length in neighborhood** |  |  |  |
| 1-5 | 1.00[1.00,1.00] | 1.00[1.00,1.00] | 1.00[1.00,1.00] |
| 6-10 | 1.78[0.71,4.48] | 0.85[0.59,1.24] | 0.93[0.50,1.74] |
| 11-15 | 4.36[1.72,11.08]^**^ | 0.88[0.54,1.41] | 2.38[1.25,4.55]^**^ |
| 16-20 | 2.73[0.93,7.94] | 1.01[0.60,1.70] | 2.38[1.18,4.82]^*^ |
| 20+ | 4.92[2.17,11.13]^***^ | 0.55[0.37,0.81]^**^ | 2.41[1.40,4.15]^**^ |
| other | 5.73[2.10,15.62]^***^ | 1.90[1.12,3.21]^*^ | 6.72[3.51,12.83]^***^ |
| **Model 2** | | | |
| **Neighborhood aesthetic quality** | 0.90[0.63,1.27] | 0.67[0.54,0.83]^***^ | 0.73[0.57,0.95]^*^ |
| **Neighborhood structural deprivation** | 1.44[1.01,2.05]^*^ | 1.13[0.90,1.42] | 1.09[0.83,1.42] |
| **Neighborhood violence** | 1.07[0.83,1.39] | 0.97[0.83,1.14] | 0.76[0.61,0.94]^*^ |
| **Neighborhood odor** | 1.10[0.86,1.41] | 1.09[0.93,1.27] | 1.26[1.03,1.53]^*^ |
| **Time in neighborhood** |  |  |  |
| Mostly away | 1.00[1.00,1.00] | 1.00[1.00,1.00] | 1.00[1.00,1.00] |
| A few days | 0.71[0.24,2.06] | 0.50[0.27,0.92]^*^ | 0.51[0.23,1.10] |
| Most days | 0.69[0.26,1.88] | 0.46[0.25,0.82]^**^ | 0.57[0.28,1.17] |
| Entire week | 0.86[0.32,2.29] | 0.65[0.36,1.16] | 0.75[0.37,1.50] |
| **Length in neighborhood** |  |  |  |
| 1-5 | 1.00[1.00,1.00] | 1.00[1.00,1.00] | 1.00[1.00,1.00] |
| 6-10 | 1.55[0.61,3.99] | 0.86[0.58,1.27] | 0.89[0.47,1.70] |
| 11-15 | 3.41[1.30,8.93]^*^ | 0.85[0.51,1.40] | 2.00[1.02,3.93]^*^ |
| 16-20 | 2.10[0.69,6.39] | 1.06[0.61,1.85] | 2.12[1.02,4.43]^*^ |
| 20+ | 3.86[1.66,8.99]^**^ | 0.54[0.36,0.82]^**^ | 2.15[1.22,3.78]^**^ |
| other | 5.75[2.02,16.39]^**^ | 1.67[0.96,2.92] | 5.89[2.98,11.63]^***^ |
| **Fruits consumption** |  |  |  |
| 1 serving | 1.00[1.00,1.00] | 1.00[1.00,1.00] | 1.00[1.00,1.00] |
| 2 servings | 0.67[0.28,1.59] | 1.03[0.65,1.64] | 0.81[0.45,1.45] |
| 3 servings | 0.95[0.39,2.30] | 1.61[0.98,2.66] | 1.26[0.67,2.36] |
| 4+ servings | 1.06[0.47,2.38] | 1.49[0.93,2.38] | 1.41[0.78,2.55] |
| other | 1.26[0.47,3.33] | 0.92[0.53,1.60] | 0.61[0.30,1.24] |
| **Vegetable consumption** |  |  |  |
| 1 serving | 1.00[1.00,1.00] | 1.00[1.00,1.00] | 1.00[1.00,1.00] |
| 2 servings | 2.11[0.67,6.67] | 0.73[0.42,1.29] | 0.67[0.33,1.35] |
| 3 servings | 1.83[0.56,5.99] | 0.89[0.51,1.54] | 0.65[0.32,1.30] |
| 4+ servings | 2.48[0.84,7.29] | 0.94[0.56,1.57] | 0.61[0.32,1.15] |
| other | 1.59[0.41,6.06] | 0.59[0.30,1.15] | 1.18[0.54,2.60] |
| **Salt at dining table** |  |  |  |
| No | 1.00[1.00,1.00] | 1.00[1.00,1.00] | 1.00[1.00,1.00] |
| Yes | 1.28[0.70,2.36] | 1.51[1.06,2.16]^*^ | 1.05[0.66,1.69] |
| **Eat away from home** |  |  |  |
| Not at all | 1.00[1.00,1.00] | 1.00[1.00,1.00] | 1.00[1.00,1.00] |
| Two and below | 0.42[0.24,0.74]^**^ | 0.91[0.64,1.30] | 0.44[0.28,0.69]^***^ |
| Three or more | 0.58[0.29,1.15] | 1.20[0.79,1.82] | 0.79[0.47,1.33] |
| Everyday | 0.11[0.04,0.37]^***^ | 0.47[0.28,0.80]^**^ | 0.38[021,0.71]^**^ |
| **Tobacco consumption** |  |  |  |
| No | 1.00[1.00,1.00] | 1.00[1.00,1.00] | 1.00[1.00,1.00] |
| Yes | 3.10[1.58,6.09]^**^ | 0.69[0.39,1.21] | 2.88[1.64,5.04]^***^ |
| **Alcohol consumption** |  |  |  |
| No | 1.00[1.00,1.00] | 1.00[1.00,1.00] | 1.00[1.00,1.00] |
| Yes | 0.80[0.45,1.44] | 1.32[0.94,1.86] | 1.10[0.70,1.73] |
| **LTPA** |  |  |  |
| Never | 1.00[1.00,1.00] | 1.00[1.00,1.00] | 1.00[1.00,1.00] |
| Rarely | 2.22[1.18,4.18]^*^ | 1.90[1.33,2.72]^***^ | 0.88[0.55,1.40] |
| 1-2 times | 1.47[0.71,3.04] | 1.75[1.16,2.65]^**^ | 1.09[0.65,1.82] |
| 3+ times | 2.59[1.20,5.60]^*^ | 2.27[1.42,3.65]^***^ | 1.89[1.09,3.30]^*^ |
| **Tap water at home** |  |  |  |
| Yes | 1.00[1.00,1.00] | 1.00[1.00,1.00] | 1.00[1.00,1.00] |
| No | 2.34[1.09,5.02]^*^ | 1.76[1.20,2.58]^**^ | 1.75[1.07,2.87]^*^ |
| **Toilet at home** |  |  |  |
| Yes | 1.00[1.00,1.00] | 1.00[1.00,1.00] | 1.00[1.00,1.00] |
| No | 0.41[0.20,0.84]^*^ | 0.75[0.52,1.10] | 0.57[0.35,0.93]^*^ |
| **Model 3** | | | |
| **Neighborhood aesthetic quality** | 0.91[0.62,1.34] | 0.73[0.58,0.92]^**^ | 0.79[0.59,1.06] |
| **Neighborhood structural deprivation** | 1.61[1.08,2.41]^*^ | 1.11[0.88,1.41] | 1.11[0.82,1.48] |
| **Neighborhood violence** | 1.21[0.92,1.59] | 0.97[0.82,1.14] | 0.83[0.66,1.04] |
| **Neighborhood odor** | 1.15[0.87,1.51] | 1.07[0.91,1.26] | 1.27[1.03,1.57]^*^ |
| **Time in neighborhood** |  |  |  |
| Mostly away | 1.00[1.00,1.00] | 1.00[1.00,1.00] | 1.00[1.00,1.00] |
| A few days | 0.78[0.25,2.45] | 0.52[0.28,0.98]^*^ | 0.51[0.23,1.15] |
| Most days | 0.76[0.26,2.22] | 0.45[0.24,0.82]^**^ | 0.51[0.24,1.09] |
| Entire week | 0.76[0.26,2.21] | 0.55[0.30,1.01] | 0.48[0.23,1.01] |
| **Length in neighborhood** |  |  |  |
| 1-5 | 1.00[1.00,1.00] | 1.00[1.00,1.00] | 1.00[1.00,1.00] |
| 6-10 | 1.01[0.38,2.71] | 0.87[0.58,1.30] | 0.64[0.32,1.26] |
| 11-15 | 1.92[0.69,5.34] | 0.85[0.50,1.45] | 1.17[0.56,2.44] |
| 16-20 | 0.99[0.30,3.25] | 1.08[0.60,1.93] | 1.04[0.47,2.32] |
| 20+ | 1.59[0.62,4.03] | 0.53[0.34,0.83]^**^ | 1.02[0.54,1.92] |
| other | 4.72[1.57,14.17]^**^ | 1.69[0.95,3.02] | 4.51[2.17,9.33]^***^ |
| **Fruits consumption** |  |  |  |
| 1 serving | 1.00[1.00,1.00] | 1.00[1.00,1.00] | 1.00[1.00,1.00] |
| 2 servings | 0.51[0.20,1.28] | 1.07[0.66,1.75] | 0.73[0.39,1.37] |
| 3 servings | 0.97[0.37,2.50] | 1.73[1.03,2.91]^*^ | 1.27[0.64,2.50] |
| 4+ servings | 0.88[0.37,2.10] | 1.62[0.99,2.66] | 1.27[0.68,2.39] |
| other | 1.23[0.43,3.52] | 0.96[0.54,1.69] | 0.61[0.28,1.33] |
| **Vegetable consumption** |  |  |  |
| 1 serving | 1.00[1.00,1.00] | 1.00[1.00,1.00] | 1.00[1.00,1.00] |
| 2 servings | 2.59[0.77,8.68] | 0.70[0.39,1.25] | 0.72[0.34,1.51] |
| 3 servings | 2.38[0.69,8.20] | 0.91[0.52,1.61] | 0.83[0.40,1.76] |
| 4+ servings | 3.14[1.01,9.75]^*^ | 0.91[0.53,1.55] | 0.69[0.35,1.37] |
| other | 1.86[0.45,7.66] | 0.55[0.28,1.09] | 1.20[0.51,2.86] |
| **Salt at dining table** |  |  |  |
| No | 1.00[1.00,1.00] | 1.00[1.00,1.00] | 1.00[1.00,1.00] |
| Yes | 1.84[0.96,3.55] | 1.55[1.07,2.25]^*^ | 1.20[0.73,1.99] |
| **Eat away from home** |  |  |  |
| Not at all | 1.00[1.00,1.00] | 1.00[1.00,1.00] | 1.00[1.00,1.00] |
| Two and below | 0.57[0.31,1.08] | 0.98[0.68,1.43] | 0.65[0.40,1.07] |
| Three or more | 1.02[0.48,2.19] | 1.50[0.97,2.33] | 1.50[0.85,2.67] |
| Everyday | 0.25[0.07,0.88]^*^ | 0.61[0.35,1.07] | 0.82[0.40,1.66] |
| **Tobacco consumption** |  |  |  |
| No | 1.00[1.00,1.00] | 1.00[1.00,1.00] | 1.00[1.00,1.00] |
| Yes | 1.86[0.87,3.99] | 0.77[0.42,1.39] | 2.26[1.20,4.26]^*^ |
| **Alcohol consumption** |  |  |  |
| No | 1.00[1.00,1.00] | 1.00[1.00,1.00] | 1.00[1.00,1.00] |
| Yes | 0.95[0.50,1.82] | 1.55[1.06,2.26]^*^ | 1.41[0.84,2.35] |
| **LTPA** |  |  |  |
| Never | 1.00[1.00,1.00] | 1.00[1.00,1.00] | 1.00[1.00,1.00] |
| Rarely | 2.20[1.11,4.38]^*^ | 2.24[1.54,3.26]^***^ | 0.97[0.59,1.61] |
| 1-2 times | 1.32[0.59,2.96] | 2.17[1.40,3.35]^***^ | 1.26[0.71,2.22] |
| 3+ times | 2.56[1.08,6.05]^*^ | 3.02[1.83,4.99]^***^ | 2.51[1.35,4.65]^**^ |
| **Tap water at home** |  |  |  |
| Yes | 1.00[1.00,1.00] | 1.00[1.00,1.00] | 1.00[1.00,1.00] |
| No | 3.00[1.19,7.56]^*^ | 1.65[1.03,2.64]^*^ | 1.99[1.02,3.88]^*^ |
| **Toilet at home** |  |  |  |
| Yes | 1.00[1.00,1.00] | 1.00[1.00,1.00] | 1.00[1.00,1.00] |
| No | 0.28[0.11,0.71]^**^ | 0.64[0.39,1.05] | 0.73[0.37,1.44] |
| **Educational level** |  |  |  |
| Tertiary education | 1.00[1.00,1.00] | 1.00[1.00,1.00] | 1.00[1.00,1.00] |
| Secondary education | 0.41[0.19,0.90]^*^ | 1.16[0.76,1.76] | 0.93[0.51,1.68] |
| Primary education | 0.43[0.16,1.13] | 1.47[0.87,2.47] | 1.38[0.68,2.79] |
| No formal education | 0.67[0.24,1.92] | 1.51[0.79,2.91] | 1.32[0.59,2.92] |
| **Employment status** |  |  |  |
| Privately employed | 1.00[1.00,1.00] | 1.00[1.00,1.00] | 1.00[1.00,1.00] |
| Government employed | 0.33[0.13,0.82]^*^ | 1.27[0.77,2.09] | 1.01[0.51,1.99] |
| Unemployed | 1.22[0.51,2.93] | 0.96[0.58,1.58] | 1.07[0.56,2.05] |
| Other | 0.41[0.20,0.84]^*^ | 1.28[0.88,1.88] | 0.74[0.44,1.25] |
| **Wealth quintile** |  |  |  |
| Poorest | 1.00[1.00,1.00] | 1.00[1.00,1.00] | 1.00[1.00,1.00] |
| Poorer | 1.96[0.70,5.48] | 1.23[0.75,2.02] | 1.14[0.57,2.28] |
| Middle | 1.68[0.45,6.29] | 0.85[0.45,1.62] | 1.07[0.42,2.70] |
| Richer | 0.69[0.14,3.45] | 1.18[0.54,2.60] | 1.99[0.65,6.09] |
| Richest | 0.90[0.16,5.00] | 0.69[0.28,1.70] | 1.53[0.44,5.34] |
| **Age** | 1.07[1.05,1.10]^***^ | 1.01[0.99,1.02] | 1.07[1.05,1.09]^***^ |
| **Gender** |  |  |  |
| Male | 1.00[1.00,1.00] | 1.00[1.00,1.00] | 1.00[1.00,1.00] |
| Female | 1.89[1.04,3.44]^*^ | 1.84[1.32,2.57]^***^ | 2.90[1.85,4.53]^***^ |
| **Marital status** |  |  |  |
| Single | 1.00[1.00,1.00] | 1.00[1.00,1.00] | 1.00[1.00,1.00] |
| In relationship | 1.78[0.48,6.60] | 0.92[0.52,1.63] | 0.41[0.12,1.35] |
| Married | 1.11[0.41,2.99] | 1.03[0.66,1.61] | 0.80[0.42,1.52] |
| Divorced/Widowed | 1.14[0.33,3.97] | 0.80[0.39,1.64] | 0.86[0.35,2.09] |
| **Religion** |  |  |  |
| Christian | 1.00[1.00,1.00] | 1.00[1.00,1.00] | 1.00[1.00,1.00] |
| Muslim | 0.97[0.50,1.90] | 1.15[0.77,1.72] | 1.39[0.84,2.31] |
| Other | 0.81[0.11,6.03] | 2.34[0.60,9.13] | 0.98[0.21,4.64] |
